# Metachronous nasopharyngeal carcinoma following sinonasal inverted papilloma – a diagnostic enigma

**DOI:** 10.1007/s00405-025-09987-5

**Published:** 2026-01-20

**Authors:** Deviprasad Dosemane, Meera Niranjan Khadilkar, Nithya Chandy, Divya Subramanian, Nayana Rao, Susana Iqbal

**Affiliations:** 1 Department of Otorhinolaryngology – Head and Neck, Kasturba Medical College Mangalore, Manipal Academy of Higher Education, Manipal, India; 2 Department of Pathology, Kasturba Medical College Mangalore, Manipal Academy of Higher Education, Manipal, India

**Keywords:** Human papilloma virus, Inverted papilloma, Nasopharyngeal carcinoma, Nasopharyngeal neoplasms, Sinonasal tract, Squamous cell carcinoma

## Abstract

**Background:**

Inverted papilloma (IP) is a benign Schneiderian epithelial tumour with a well-recognized potential for recurrence and malignant transformation, most commonly to squamous cell carcinoma (SCC). Carcinoma arising in the nasopharynx after prior IP is exceedingly rare, and distinguishing malignant conversion from a metachronous nasopharyngeal carcinoma (NPC) poses a diagnostic challenge.

**Case report:**

We report a rare, interesting case of a nasopharyngeal lesion with histopathological features of non-keratinizing SCC in a patient previously treated surgically for sinonasal IP.

**Conclusion:**

Differentiating malignant transformation of IP from a metachronous malignancy is complex. In this case, the short interval between diagnoses, anatomical separation, and absence of Schneiderian features favour interpretation as a metachronous NPC, highlighting the importance of continued surveillance and prompt evaluation of new symptoms in patients treated for IP.

**Supplementary Information:**

The online version contains supplementary material available at 10.1007/s00405-025-09987-5.

## Introduction

 Inverted papillomas (IPs) are benign tumours of the Schneiderian membrane, typically arising from the nasal cavity or paranasal sinuses, most often in middle-aged men. They are pre-malignant, with high recurrence and potential for local invasion, typically originating from the lateral nasal wall or ethmoid sinus [1, 2]. Occurrence of IP in the nasopharynx is rare [3]. Early recurrence of IP has been reported within 3–5 years of surgery in 8.5–11% and late recurrence in 5–15 years post surgery in 44% cases; risk factors include incomplete surgical clearance, high-risk Human Papilloma Virus (HPV) infection, high mitotic index, hyperkeratosis, and severe epidermal dysplasia [4]. Malignant transformation occurs in about 7–10% of cases, most commonly to squamous cell carcinoma (SCC) [5]. SCC may manifest concurrently with the primary IP (synchronous) in 7.1% cases, or after preceding treatments for benign IP (metachronous) in 3.6% cases [6].

Nasopharyngeal carcinoma (NPC) is an aggressive malignant tumour of head and neck arising from epithelial cells lining the nasopharyngeal mucosa, with Fossa of Rosenmüller being the epicentre. It has a bimodal age distribution with peaks in third and seventh decades of life. Besides environmental factors, genetics and Epstein Barr Virus (EBV) infection, HPV is predominantly associated with a risk of keratinizing or differentiated non-keratinizing NPC in inhabitants of non-endemic regions [7].

This report presents an unusual case of nasopharyngeal carcinoma diagnosed in a patient with prior surgical excision for inverted papilloma.

## Case report

**Fig. 1:**
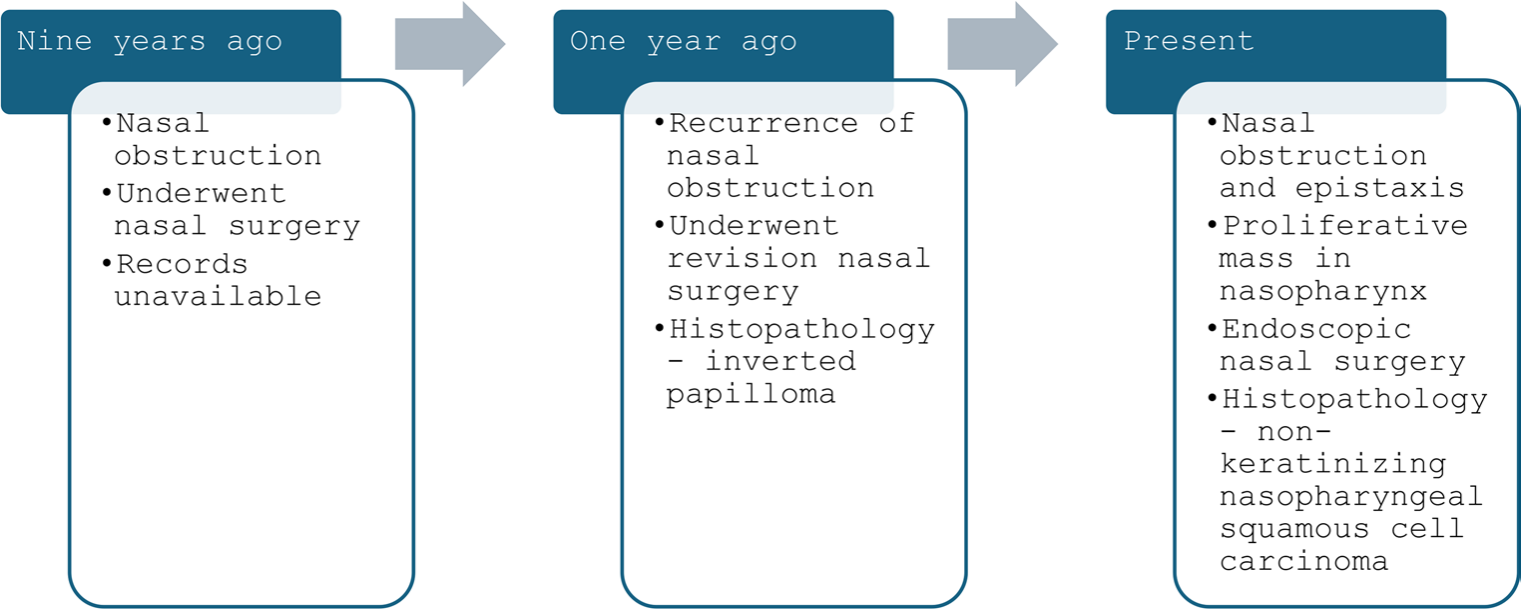
Timeline of events

A 31-year-old woman presented with a one-week history of increasing right-sided nasal obstruction and blood-tinged discharge. She had a history of nasal obstruction nine years ago, treated surgically at another center, the records of which were unavailable. One year ago, she underwent revision surgery for recurrent symptoms; histopathology of the specimen showed features consistent with inverted papilloma. Current symptoms of obstruction and recent-onset epistaxis led to evaluation at our center (Fig. 1). Endoscopic examination showed left sided septal deviation and a proliferative mass with slough-covered areas, occupying the nasopharynx and obstructing both fossae of Rosenmuller (FoR).

**Fig. 2:**
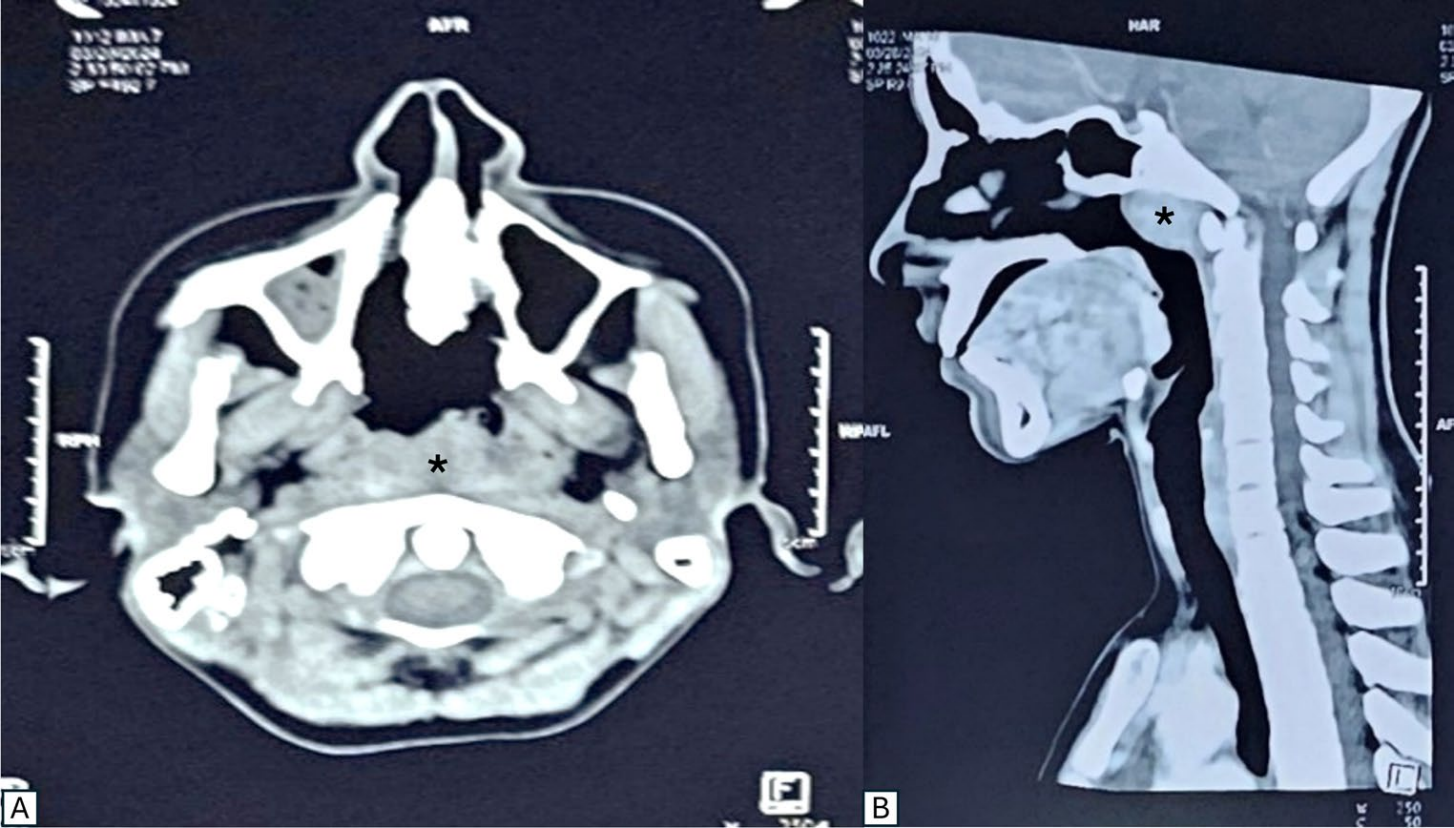
Computed tomography image (**A**) coronal section and (**B**) saggital section showing a well-defined, lobulated, heterogeneously enhancing lesion (asterisk) arising from the posterosuperior nasopharyngeal wall

**Fig. 3:**
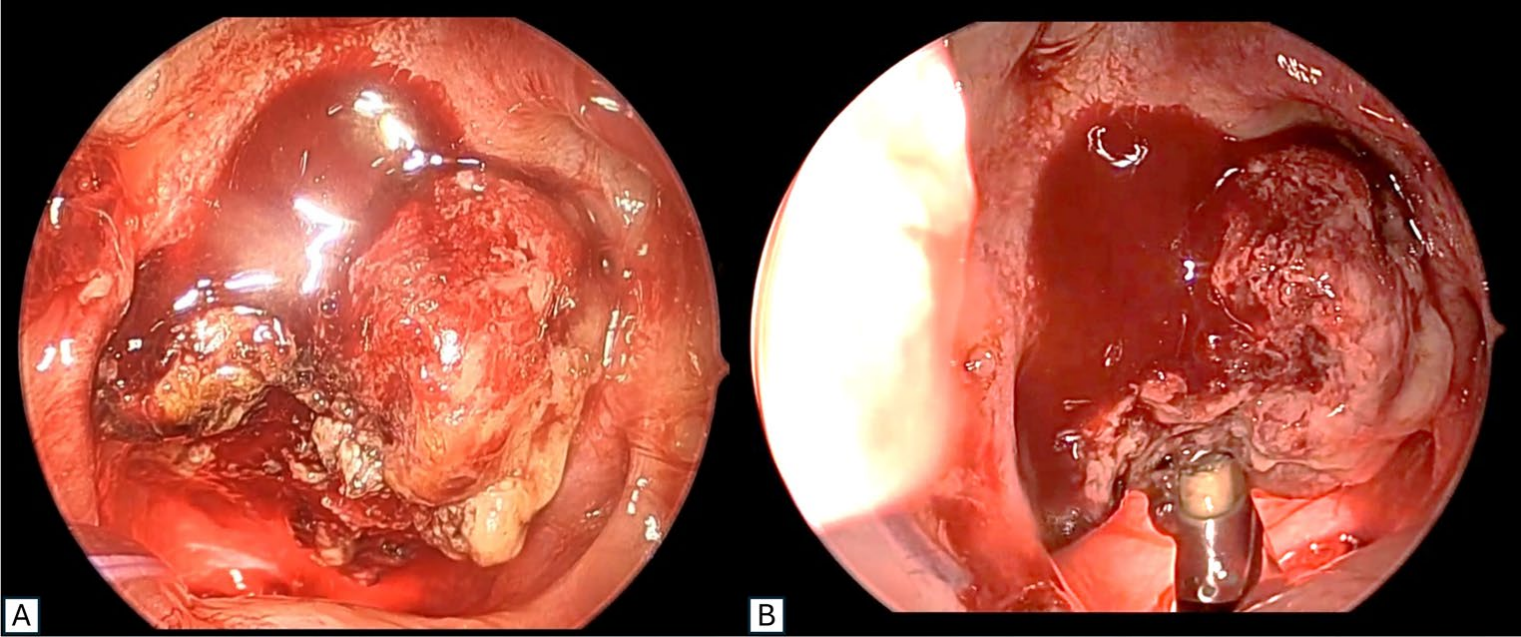
Intraoperative endoscopic photographs of the lesion (**A**) before excision (**B**) during coblator-assisted excision

Contrast-enhanced Computed Tomography (CECT) of neck showed a well-defined, lobulated, heterogeneously enhancing lesion (1.8 × 2.2 × 2.5 cm) arising from the posterosuperior nasopharyngeal wall, partially obliterating the nasopharyngeal airway, extending into the nasal choanae, bilateral FoR, and abutting the clivus, and sparing the prevertebral space (Fig. 2). The patient underwent endoscopic excision under general anaesthesia, with multiple biopsies taken prior to complete coblation-assisted removal (Fig. 3). Postoperative recovery was uneventful; follow-up endoscopy showed a clear nasopharynx with no residual disease.

**Fig. 4:**
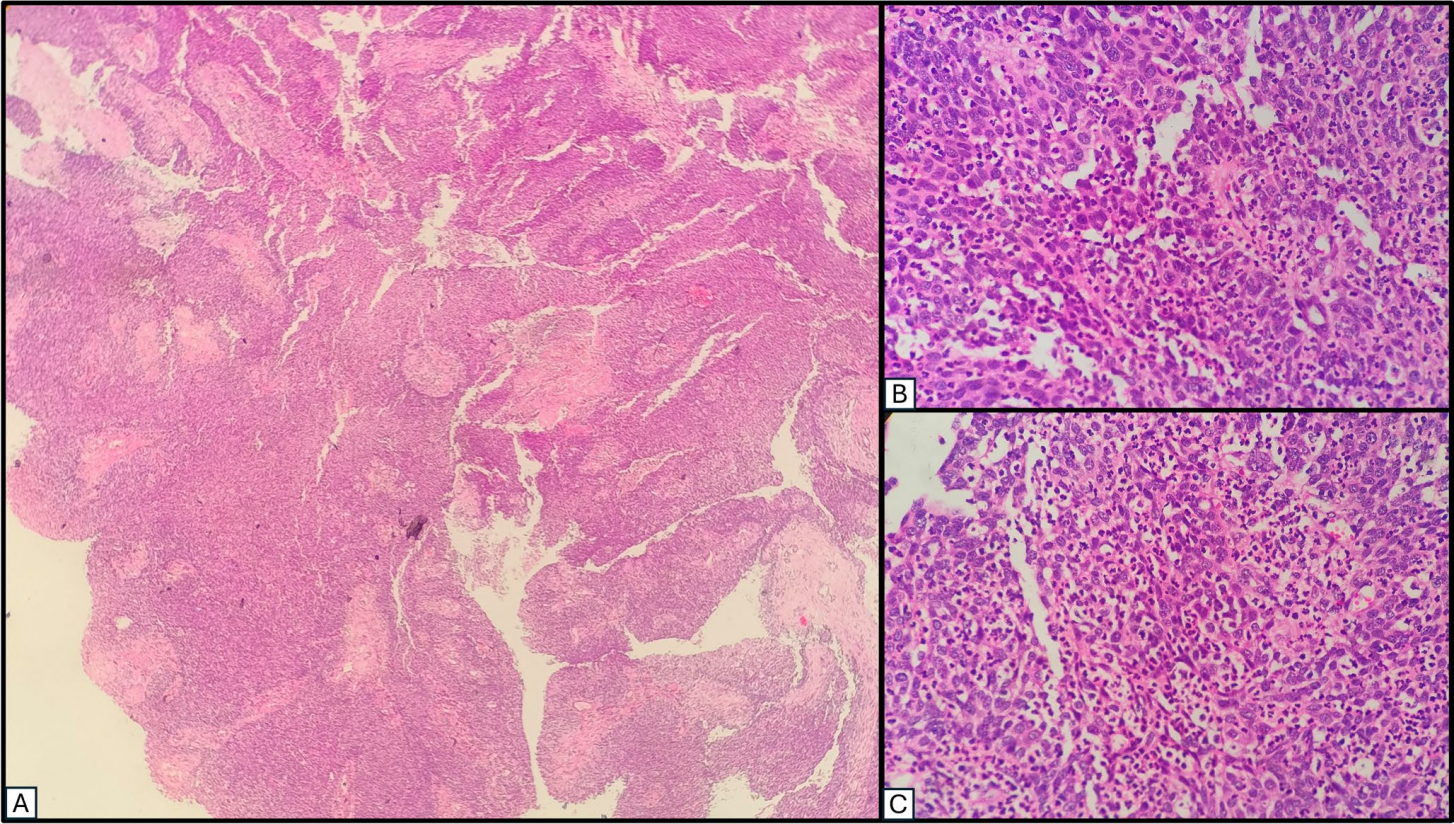
Histopathology photomicrograph – haematoxylin & eosin stain (**A**) ×40x – showing a polypoidal lesion with endophytic growth pattern and invaginations of stratified squamous epithelium into the underlying stroma with a fibrovascular core and chronic inflammatory infiltrate (**B**) ×400x – showing epithelial dysplasia with loss of polarity, nuclear pleomorphism, and increased nuclear–cytoplasmic ratio with nests and sheets of atypical squamous cells in the underlying stroma, and desmoplastic reaction (**C**) ×400x – showing infiltrating squamous cells with keratin pearl formation and prominent nucleoli

Histopathology revealed an invasive tumour with predominantly exophytic papillary architecture, composed of moderately pleomorphic squamous cells with minimal atypia and focal spindle cell arrangement (Fig. 4). A dense neutrophilic infiltrate was present. Immunohistochemistry showed p16 and p40 positivity, with EBV negativity. Findings were consistent with non-keratinizing nasopharyngeal squamous cell carcinoma. The patient was referred for chemoradiation.

## Discussion

While etiopathogenesis of IP remains debated, factors such as chronic inflammation, irritant exposure, and viral infections have been implicated [8, 9]. IPs typically arise from the lateral nasal wall, with possible extension into adjacent sinuses or, rarely (3%), into the nasopharynx [10]. Primary IPs of the nasopharynx are extremely uncommon [11–19]. While typical IPs follow a well-characterized course, our case is notable for the rare development of NPC in a patient with recurrent IP.

Malignant transformation of IP has been reported in 5–27% cases, most commonly to squamous cell carcinoma (SCC) [1]. Such synchronous SCC occurs due to dysplasia of sinonasal epithelium of IP. Distinguishing carcinoma arising from IP from low-grade papillary Schneiderian carcinoma (LGPSC) is essential, as the latter shows infiltrative papillary growth without atypia, whereas non-keratinizing squamous cell carcinoma (NKSCC) shows higher atypia and invasive features. Immunohistochemistry aids differentiation, with increasing Ki-67 and p53 expression from IP to LGPSC to NKSCC [19]. The histologic criteria for defining malignant conversion are not well established, and malignant foci may constitute only a fraction of the lesion [1]. We found records confirming the initial pathology to be IP in our patient, raising the possibility that no overt malignancy was present one year ago.

Metachronous SCC may arise at the site of prior IP excision. The average interval between IP and onset of a metachronous SCC is around 63 months [20]. Contrastingly, our patient developed NPC within one year of IP excision, indicating an aggressive progression of the disease. Furthermore, metachronous cancers after IP more commonly affect the maxillary sinus and nasal cavity, and rarely the nasopharynx [20]. Only 4 cases of NPC arising from a previous IP have been reported, making our case noteworthy [20–23].

The virological profile in this case—HPV positivity with EBV negativity—aligns with patterns observed in non-endemic NPC, where keratinizing and non-keratinizing SCCs may be HPV-associated. While high-risk HPV, particularly type 18, has been implicated in malignant conversion of sinonasal IP [9], EBV has not been shown to play a role in such transformation outside endemic regions [23]. Nevertheless, p16 immunohistochemistry, used as a surrogate marker of HPV infection, has inherent limitations, including false positivity and lack of direct correlation with viral DNA integration [24]. Furthermore, HPV genotyping was not performed in this case, limiting precise characterization of viral oncogenesis.

Interpretation is limited by the absence of the original histopathological material from the previously excised IP, which precludes direct morphologic comparison and confirmation of a pathogenetic continuum. Although a definitive causal relationship cannot be established, the diagnosis of NPC one year after IP excision, its occurrence at an anatomically distinct site, and the lack of residual Schneiderian architecture or precursor dysplasia in the nasopharyngeal lesion collectively suggest a metachronous SCC rather than malignant transformation of the original IP. Long-term surveillance and prompt evaluation of new symptoms is essential in such cases of IP to aid in early diagnosis of malignancy and management.

## Conclusion

Differentiating malignant transformation of inverted papilloma from a metachronous malignancy can be challenging. In this case, the one-year interval, anatomical separation, and lack of residual Schneiderian features support interpretation as a metachronous nasopharyngeal carcinoma rather than transformation of the original lesion. This emphasizes the need for continued surveillance and prompt evaluation of new symptoms in patients previously treated for inverted papilloma.

## Supplementary Information

Below is the link to the electronic supplementary material.

Supplementary Material 1 (DOCX 55.1 KB)
