# Supplementary material for: Metachronous nasopharyngeal carcinoma following sinonasal inverted papilloma – a diagnostic enigma
Source: Eur Arch Otorhinolaryngol. 2026 Jan 20;283(4):2591–5. doi: 10.1007/s00405-025-09987-5 (PMC13053405; doi:10.1007/s00405-025-09987-5)
Supplement: Supplementary file 1 — Supplementary Material 1 (DOCX 55.1 KB) [file 405_2025_9987_MOESM1_ESM.docx]

**Table 1 – Case profile of IP in nasopharynx**

| **Case History** | **Clinical examination** | **Imaging Findings** | **Treatment** | **Follow-up** |
| --- | --- | --- | --- | --- |
| 1. 42-year-old male – 1-week-long sore throat, hoarseness, no nasal complaints (Radcliffe, 1953) | Papillomatous mass on posterior pharyngeal wall extending from basisphenoid to 1 inch above epiglottis | X-ray paranasal sinuses – normal | Transverse palatine incision to expose posterior pharyngeal wall, mass excised using diathermy | Severe pain, neck stiffness, occipital headache, dysphagia postoperatively, left-sided Horner’s syndrome, progressive right nystagmus, spitting out sequestered pieces of anterior arch of atlas – but postnasal space, nasal cavities disease-free |
| 2. 44-year-old man - left OME (Wolff et al., 1980) | 1x1cm soft, protuberant bluish mass blocking left ET orifice | Polytome tomography – non-invasive lesion | Subperiosteal excision with wide margins via midline soft palate split approach | No recurrence at 30 months |
| 3. 58-year-old male – referred for recurrence of IP, post-right intranasal polypectomy and ethmoidectomy 14 months prior (Astor et al., 1985) | Papillomatous growth in right middle meatus,  second lesion in nasopharynx (intraoperative) | Not mentioned | Medial Maxillectomy, second lesion noted intraoperatively arising from lateral nasopharyngeal wall, excised with wide margins | Disease free at 5 years |
| 4. 68-year-old man – chronic smoker, referred for nasopharyngeal mass extending to oropharynx, allergic to penicillin (Low et al., 2002) | Polypoidal mass arising from left postnasal space at tubal elevation | Not mentioned | Endoscopic transnasal + transoral excision with 0.5mm margins | No recurrence at 2 years |
| 5. 52-year-old man – 4-week-long right sided decreased hearing, no nasal complaints (Fageeh & Al-Shraim, 2013) | Small, fleshy polypoidal lesion arising from posterior wall of nasopharynx near right torus, serous collection in middle ear | CECT nasopharynx and neck – normal | Transnasal endoscopic surgery – submucosal dissection, sparing of torus tubarius + right ear grommet insertion | No recurrence at 12 month follow-up with fiberoptic examination |
| 6. 71-year-old woman – rustling tinnitus in right ear aggravated on swallowing and phonation, no nasal obstruction (Kishikawa et al., 2014) | DNE – large pharyngeal mass arising from right choana, fully occupying nasopharynx, pedicle just anterior to sphenoid orifice.  PTA – normal.  Sonotubometry – complete block of right ET  Acoustic rhinomanometry – severe right nasal obstruction | MRI – T2 hyperintense, gadolinium-enhancing tumour confined to nasopharynx, no invasion into sphenoid sinus or skull base | Endoscopic removal of lower part of IT, MT shifted, periosteal incision around pedicle, periosteum dissected from bone to excise pedicle, tumour delivered orally | No recurrence at 1.5-year follow-up on clinical examination and imaging |
| 7. 77-year-old male – 1-month-long left-sided hearing loss, sore throat (Ohki, 2015) | Left – irregular mass in FoR extending to anterior nasopharynx, ET.  Right – irregular mass in nasal cavity involving IT, floor, septum | Enhanced T1-weighted MRI – high-intensity signals in left nasopharynx, including FoR, parapharyngeal space, right IT | Conservative management – Clarithromycin 400 mg and Carbocisteine 750mg for 2 weeks (at patient request) | No tumour proliferation over 3-month follow-up |
| 8. 7.5-year-old girl – persistent right OME, snoring (Vlastarakos et al., 2016) | Symmetrically enlarged adenoids | Not mentioned | Cold steel adenoidectomy + grommet insertion | 3 months later – MRI normal  6 months later – examination of nasopharynx under GA - adenoid at left choanae, excised; biopsy – IP – disease free at 1 year |
| 9. 53-year-old woman – recurrent right ear purulent discharge, hearing loss, no nasal obstruction (Yang et al., 2021) | Growth in right EAC and left part of nasopharynx | MRI – changes in right parapharyngeal space, nasopharynx, and ethmoid sinus 🡪 recurrence | Surgical resection | Recurrence of both lesions, 3 revision surgeries with similar histopathology.  Three years later nasal obstruction, headache, giddiness;  Biopsy – malignant transformation of IP – unfit for surgery, Anlotinib + IMRT 🡪 successful remission |

^CECT – contrast enhanced computed tomography, DNE – diagnostic nasal endoscopy, PTA – pure tone audiometry, ET – Eustachian tube, MRI – magnetic resonance imaging, IT – inferior turbinate, MT – middle turbinate, FoR – fossa of Rosenmuller, OME – otitis media with effusion, EAC – external auditory canal, IP – inverted papilloma, IMRT - intensity modulated radiation therapy^

**Table 2 – Case profile of NPC arising from IP**

| **Case History** | **Clinical examination** | **Imaging Findings** | **Treatment** | **Follow-up** |
| --- | --- | --- | --- | --- |
| 1. 49-year-old male asymptomatic patient (Sulica et al., 1999) | Nasopharyngeal lesion noted on workup for SCC of cervical node | Not mentioned | Wide field radiotherapy | 30 month follow up – death |
| 2. 81-year-old woman – post-excision of sinonasal IP, followed two months later by hearing loss, pulsatile tinnitus, right blood-stained otorrhoea with malignant transformation (Liu et al., 2013) | Recurrence of lesion extending to nasopharynx, involving right EAC and soft palate | CT temporal bone – soft tissue lesion in nasopharynx adjacent to ET, with bony EAC erosion, extending into right middle ear cavity and mastoid air cells | Repeat biopsy – invasive NKSCC papillary type, p16 and p63 positive 🡪 Palliative radiotherapy | Not mentioned |
| 3. 61-year-old Caucasian male with recurrent IP with malignant conversion to SCC with left neck mass (Sharbel et al., 2019) | Not mentioned | MRI & PET-CT – nasopharyngeal recurrence, clival erosion, left cervical & retropharyngeal node avidity | Endoscopic transpterygoid nasopharyngectomy + resection of clivus & ET with nasoseptal flap reconstruction, staged bilateral & retropharyngeal neck dissection + adjuvant therapy | Histopathology - poorly differentiated invasive NKSCC, p16 and EBV negative.  Three months post-surgery – cervical vertebral fracture due to osteoradionecrosis + recurrence 🡪 death |
| 4. 31-year-old woman – right nasal obstruction, discharge and bleed for 5 months with history of removal of? viral papilloma on right side of nose, biopsy – high grade dysplasia carcinoma in situ, possibly arising from IP (Flach et al., 2020) | Physical examination – unremarkable.  Flexible rhinoscopy – normal on left, difficult view on right | CT – soft tissue mass filling right nasal cavity, opacification of right maxillary, ethmoid, frontal sinuses, thinning of medial wall of right maxillary sinus | Endonasal full thickness resection of site of origin, right MMA, uncinectomy, limited anterior ethmoidectomy 🡪 biopsy – papillary carcinoma in situ | Two further endonasal resections over 2 years 🡪 at 2.5 years post-surgery – skip lesion in nasopharynx – surgical resection – biopsy showed NPC in situ arising from IP 🡪 at 3 years – solitary exophytic lesion in ET orifice – invasive poorly differentiated carcinoma extending into skull base and prevertebral space 🡪 palliative chemotherapy |

^IP – inverted papilloma, CT – computed tomography, MMA – middle meatal antrostomy, NPC – nasopharyngeal carcinoma, ET – Eustachian tube, SCC – squamous cell carcinoma, MRI – magnetic resonance imaging, PET – positron emission tomography, NKSCC – non-keratinizing squamous cell carcinoma, EBV – Epstein Barr Virus, EAC – external auditory canal^
